# Effects of 4-methylimidazole on cerebral glutamate decarboxylase activity and specific GABA receptor binding in mice

**DOI:** 10.1080/15376510802488173

**Published:** 2009-06-30

**Authors:** Tore Sivertsen, Ann-Kristin Nygaard, Gro Mathisen, Frode Fonnum

**Affiliations:** 1 Department of Production Animal Clinical Sciences, Norwegian School of Veterinary Science, Oslo, Norway; 2 Norwegian Defence Research Establishment, Kjeller, Norway; 3 Department of Pharmacy, University of Oslo, Oslo, Norway

**Keywords:** Ammoniated forage poisoning, Convulsions, GABA, Glutamate decarboxylase, 4-Methylimidazole

## Abstract

4-Methylimidazole (4MeI) is a tremorogenic and convulsive agent of concern both in human and veterinary toxicology. The in vitro effects of 4MeI (5 μM–20 mM) on cerebral glutamate decarboxylase (GAD) activity and (in concentrations up to 50 mM) on binding of [^3^H]GABA to cerebral GABA receptors were tested in brain tissue from B6D2 mice. The effects of 1-methylimidazole (1MeI), 2-methylimidazole (2MeI), 4-methylhydroxy-imidazole (4MeOHI), imidazole-4-aceticacid (4AcI) (all in concentrations of 5–20 mM) and imidazole (20 mM) on GAD activity were also tested. In addition, the effect of a lethal dose of 4MeI (250 mg/kg ip) to B6D2 mice in vivo on the postmortem concentrations of γ-aminobutyric acid (GABA) and glutamate in their brains were measured. In all experiments, student's *t*-test was used for statistical comparison. 4MeI in concentrations of 2 mM and above did inhibit GAD activity significantly in vitro, but glutamate and GABA concentrations in mouse brains after lethal 4MeI poisoning were not significantly different from control values. The effect of 2MeI on GAD activity was stronger than the effect of 4MeI. Binding of [^3^H]GABA to cerebral GABA receptors in vitro was significantly inhibited only at 4MeI concentrations of 5 mM and above. The results indicate that neither inhibition of GABA synthesis nor competitive inhibition of the binding of GABA to its receptors are likely mechanisms for the excitation and convulsions seen in 4MeI poisoning in animals.

## Introduction

The toxicity of 4-methylimidazole (4MeI) is a matter of concern both in human and veterinary toxicology (Morgan 2004; Chan et al. 2008). The substance was first identified as a tremorogenic and convulsive agent by Nishie et al. (1969), after the observation of violent signs of CNS toxicity in cattle fed ammoniated molasses (Wiggins 1956). 4MeI induces the same signs of toxicity whether it is given orally or parenterally. Nishie et al. (1969) determined the po LD_50_ to 370 mg/kg, and the ip LD_50_ to 165 mg/kg.

The mechanism behind the excitatory and convulsive effects of 4MeI is not known. The effects can be counteracted by chlordiazepoxide and by sodium phenobarbital (Nishie et al. 1969). Both these drugs are considered to exert their main effect via GABA_A_ receptors in the brain (Charney et al. 2006). Similarity of the clinical signs induced by 4MeI and by known inhibitors of GABA synthesis such as mercaptopropionic acid and mercaptobutyric acid (Lamar 1970; Karlsson et al. 1974) does also hint at the GABA system as a possible target for 4MeI toxicity. In experimental 4MeI poisoning, sudden appearance of dramatic convulsions after a dose-dependent latent period is characteristic (Sivertsen and Müller 1999). This picture could be consistent with a build-up or depletion mechanism in the mouse brain, such as the depletion of cerebral GABA levels induced by inhibitors of cerebral GAD activity (Karlsson et al. 1974).

On this background, the present study was designed to clarify if 4MeI has sufficient effect on cerebral GABA synthesis or on specific GABA receptor binding to explain the excitatory and convulsive effects of the substance. The study was divided into three parts: an investigation of the effects of 4MeI on mouse brain glutamate decarboxylase (GAD) activity in vitro, an investigation of the effects of acute 4MeI poisoning on the immediate post-mortem GABA and glutamate concentrations in the mouse brain, and an investigation of the effects of 4MeI on specific [^3^H]GABA receptor binding in mouse brain synaptosomal membranes in vitro.

## Materials and methods

### Animals

All animals were B6D2 mice (Norwegian Institute of Public Health, Oslo). They were caged in groups of 10 with free access to food and tap water, with a 12-hour light/day cycle, a relative humidity of 50%, and a room temperature of 25°C. Ca 60 animals were used altogether. The experimental work was conducted at and approved by the Norwegian Defence Research Establishment at Kjeller, Norway. The experimental animals were handled in accordance with institutional and national guidelines for animal research.

### Effect of 4MeI on GAD activity

GAD can be obtained in a soluble form after hypotonic shock of nerve terminals (Fonnum 1968). B6D2 mice were euthanized by cervical dislocation. The brain was taken out and homogenized in 20 volumes of 0.32 M sucrose. The homogenate was centrifuged at 700 ×g for 10 minutes. The pellet was discarded and the supernatant centrifuged at 13 000 ×g for 30 minutes. The pellet from this centrifugation was shocked with cold, distilled water, re-homogenized and centrifuged at 20 000 ×g for 30 minutes. The supernatant, hereafter referred to as brain enzyme extract, was used for the GAD assays.

GAD activity was assayed by a modified version of the radiochemical method described by Albers and Brady (1959) and Fonnum et al. (1970). The brain enzyme extract (2 μl) was mixed with varying concentrations of 4MeI and with a prepared incubation solution, to a total volume of 4 μl in each test tube. The final mixture contained 11 μCi/ml Na L-[1-^14^C]-glutamate, 9.2 mM Na L-glutamate, 9.3 μM pyridoxal phosphate, 0.84 mM dithiotreitol, 18.9 mM NaH_2_PO_4_ buffer, and 0.24% Triton X-100. The final pH was 6.5. The final 4MeI concentrations used were 5 μM, 50 μM, 100 μM, 500 μM, 1 mM, 2 mM, 5 mM, and 10 mM.

The assay was run four times with each 4MeI concentration, with controls (without 4MeI) and with blanks (without brain enzyme extract). Through previous experiments it was confirmed that the activity recorded was linear with the amount of GAD enzyme (Fonnum et al. 1970). The whole experiment was repeated three times, and the results calculated as activity in percent of controls. Thereafter, GAD activity assays were repeated with the following variations in experimental conditions: with 4MeI concentration fixed at 5mM, the assays were run with Na L-glutamate concentrations of 9.2 mM, 2.3 mM, and 575 μM. With 4MeI concentration at 5 mM and Na L-glutamate at 2.3 mM, the assays were also run with pyridoxal phosphate concentrations of 9.3 μM, 4.7 μM, 2.3 μM, 0.9 μM, and 0 μM. The assay was run at least six times for each set of concentrations.

With Na-L glutamate concentration at 2.3 mM and pyridoxal phosphate at 9.3 μM, a comparative study of the effects of the following substituted imidazoles were carried out, in concentrations from 5–20 mM (minimum of six repeats per substance and concentration): 4MeI, 1-methyl imidazole (1MeI), 2-methyl imidazole (2MeI), 4-hydroxymethyl imidazole (4MeOHI), and imidazole-4-acetic acid (4AcI). In addition, the effect of 20 mM unsubstituted imidazole was tested.

### Concentrations of GABA and glutamate in mouse brain after lethal poisoning with 4MeI

Ten B6D2 mice, weighing 31–39 g, were used. Mouse no. 1 was given 250 mg 4MeI/kg body weight ip, and mouse no. 2 was given an equal volume of 0.9% NaCl solution ip immediately afterwards, to serve as control. When mouse no. 1 developed violent convulsions and died (Sivertsen and Müller 1999), mouse no. 2 was immediately euthanized by cervical dislocation. The same procedure was repeated with mice no. 3 and 4, no. 5 and 6, etc.

The heads of the dead and euthanized mice were immediately cooled in liquid N_2_, and the brains were dissected out on ice. Each brain was homogenized in 6 ml 2.5% trichloroacetic acid, with 0.5 mM α-aminoadipic acid added as internal standard. The homogenate was centrifuged for 10 minutes at 15,000 rpm. The supernatant was extracted three times with ethyl ether to remove non-polar substances. Concentrations of glutamate and GABA in the supernatant were measured by the method of Lindroth and Mopper (1979), as modified by Sandberg and Corazzi (1983), applying precolumn o-phtaldialdehyde derivatization. The samples were injected on a Varian 500LC apparatus with a reversed-phase Supelcosil LC-18-DB column, a mixture of 50 mM NaH_2_PO_4_ buffer (pH 5.25) and methanol as mobile phase, and a CMA/280 fluorescence detector. Gradient eluation was used, with the amount of NaH_2_PO_4_ buffer changing from 75% to 25% over 20 minutes (Paulsen and Fonnum 1988). The protein concentration in the pellet was measured by reaction with Folin-Ciocalteu reagent and analysis with a spectrophotometer at 750 nm, according to Lowry et al. (1951). GABA and glutamate concentrations in the mouse brains were calculated as μMol/mg protein.

### Effect of 4MeI on [^3^H]GABA binding to cerebral GABA receptors

The effect of 4MeI on sodium independent binding of [^3^H] GABA to synaptosomal membranes from mouse brain was investigated with a modified version of the method described by Hill et al. (1984) and Naalsund and Fonnum (1986). Brains from B6D2 mice were homogenized in 20 volumes of 0.32 M sucrose and centrifuged at 1000×g for 10 minutes, and the pellet was washed once. The combined supernatants were then centrifuged at 20 000 ×g for 20 minutes. The pellet from this centrifugation was resuspended to the same volume with cold, distilled water to rupture membrane vesicles, and kept on ice for 1 hour. After centrifugation at 8000 ×g for 20 minutes the supernatant was gently decanted, and the upper layer of the pellet was rinsed off and added to the supernatant. These combined supernatants were recentrifuged at 48 000 ×g for 20 minutes, and the pellet stored frozen at −20°C prior to use. For the receptor binding assay, the membranes were thawed, resuspended in TrisHCl buffer (50 mM, pH 7.4), and incubated for 45 minutes at room temperature before centrifugation (8500 ×g for 10 minutes). The membrane suspension was further washed three times and incubated with the Tris-buffer for 15 minutes at room temperature between each centrifugation, to remove all endogenous ligand. The final pellet was resuspended to give a protein concentration of ca. 100 μg per assay.

Binding of [^3^H]GABA (final concentration 25 nM with a specific activity of 40 Ci/mmol) was assayed in the absence or presence of 1 mM unlabeled GABA, to determine the specific transmitter binding. Varying amounts of 4MeI (0, 5 μM, 50 μM, 100 μM, 500 μM, 1 mM, 2 mM, 5 mM, and 50 mM final concentration) were added. All assay mixtures were diluted to a final volume of 500 μl with TrisHCl, before incubation for 60 minutes at 0°C. The membranes were collected on filters with a Skatron cell harvester, the filters were transferred to scintillation cups, dissolved in 10 ml Filter Count counting liquid and counted in a Packard 2200CA liquid scintillation analyzer. Three parallel assays without and two with unlabeled GABA were run for each 4MeI concentration. The complete series of assays was repeated four times, and the results calculated as GABA receptor binding in per cent of the mean binding without 4MeI.

### Statistical analysis

In all comparisons, student's *t*-test was used for statistical evaluation. Unless stated otherwise, the limit of statistical significance was set at *p* < 0.05.

## Results

In the first set of experiments on cerebral GAD activity, 4MeI concentrations from 5 μM to 1 mM did not reduce GAD activity significantly, compared to control values. With 2 mM 4MeI the GAD activity was reduced to 85.4% of controls (SD 4.9%), with 5 mM to 77.9% (SD 7.1%), and with 10 mM 4MeI to 61.7% of controls (SD 10.4%). All these reductions were statistically significant (*p* < 0.05).

With the 4MeI concentration set at 5 mM, neither variation of pyridoxal phosphate concentrations in the assay mixture from 0.9–9.3 μM nor variation of glutamate concentrations from 575 μM to 9.2 mM did affect the influence of 4MeI on GAD activity significantly.

**Figure 1:**
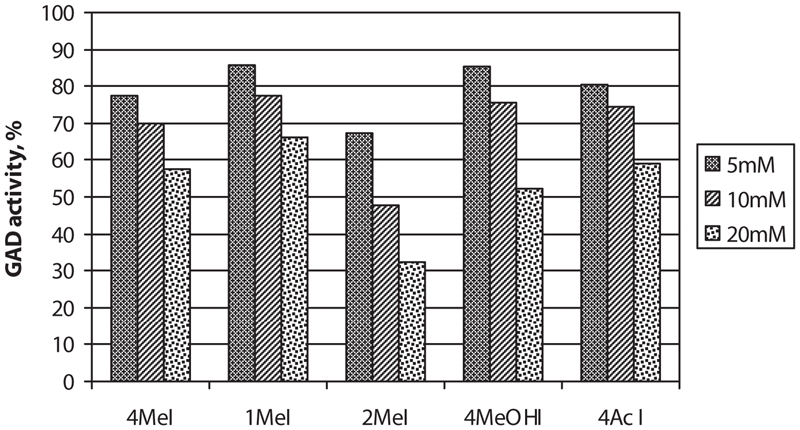
Effect of different concentrations of 4-methylimidazole (4MeI), 1-methylimidazole (1MeI), 2-methylimidazole (2MeI), 4-methylhydroxy-imidazole (4MeOHI), and imidazole-4-acetic acid (4AcI) on mouse brain glutamate decarboxylase (GAD) activity in vitro: Per cent of control activity. Mean values, *n* ≥ 6. All means are significantly different from control values (*p* < 0.01).

In the comparative experiment, all the substituted imidazoles tested reduced the GAD activity significantly (*p* < 0.01) in concentrations from 5–20 mM. In equal concentrations, 2MeI had stronger effect on GAD activity than 4MeI (Figure 1). Twenty mM imidazole also reduced the GAD activity (*p* < 0.01), but with somewhat less effect (83.1% of control) than the substituted imidazoles.

After ip injection of 250 mg 4MeI/kg, all the mice tested developed sudden convulsive seizures, after a lag period varying from 3 minutes 50 seconds to 6 minutes 50 seconds. They died in a few seconds after the onset of seizures. Average postmortem cerebral GABA concentrations in the poisoned mice were 22.1 μMol (SD 1.8 μMol) per mg protein, compared to 20.3 μMol (SD l.0 μMol) per mg protein in the control mice. The average cerebral glutamate concentrations were 119.7 μMol (SD 9.8 μMol) per mg protein in the poisoned mice, and 114.6 μMol (SD 8.2 μMol) per mg protein in the controls. None of the differences between poisoned and control mice were statistically significant.

4MeI concentrations from 5 μM to 2mM did not have a significant effect on sodium independent binding of [^3^H]GABA to synaptosomal membranes from mouse brain. With 5 mM 4MeI present the [^3^H]GABA binding was significantly (*p* < 0.01) reduced; to 63.4% (SD 7.2%) of control values. With 50 mM 4MeI the binding was reduced to 23.9% (SD 3.7%) of controls (*p* < 0.01).

## Discussion

Since the original report of Wiggins (1956), poisoning of ruminants by ammoniated forage has been observed worldwide. The poisoning is characterized by sudden episodes of agitated confusion, hyperexcitability, tremors, and convulsions (Morgan 2004). 4MeI was for many years generally accepted as the main etiologic agent in this poisoning syndrome (Osweiler 1996). Findings of very low concentrations of 4MeI in the feed and plasma of experimentally poisoned animals have complicated the picture (Sivertsen et al. 1993; Morgan 2004). Still, 4MeI is up to now the strongest and most abundant convulsive agent identified in poisonous ammoniated forage (Müller et al. 1998), and the clinical signs of experimental 4MeI poisoning are indistinguishable from those of poisoning with ammoniated feed (Kristensen et al. 1991). Clonic seizures and hyperactivity have also been observed after oral exposure to 4MeI in long-term feeding studies in rats (Chan et al. 2008).

In lethal doses, the effect of 4MeI in mice is characteristic and dramatic. Shortly after parenteral administration of 250 mg/kg 4MeI the treated mouse may show moderate signs of CNS excitation: Raised tail, slight tremors, small squeaks and jumps, and repeated grooming of the muzzle with its fore paws. After a period of 3–7 minutes, the mouse goes suddenly into violent convulsions, similar to those described by Gale (1992) as running-bouncing clonic seizures. Within a short time, usually a few seconds, the seizures end in a typical tonic extensor convulsive spasm (Gale 1992) and death (Sivertsen and Müller 1999). This toxicological picture requires that the number of animals used in in vivo studies should be kept as low as possible (Sivertsen and Müller 1999). Accordingly, the number of mice given toxic 4 MeI doses in the present study was restricted to five. From a mechanistic point of view, the clinical picture of acute 4MeI poisoning does in our opinion indicate that 4MeI interferes with one or more of the major transmitter systems in the brain (Gale 1992; McNamara 2006).

In the present study, 4MeI was found to have a dose-dependant inhibitory effect on GAD activity in vitro, statistically significant at concentrations of 2 mM or above. An average whole brain 4MeI concentration of 2.2 mMol/kg has been found in mice after lethal poisoning with 250 mg/kg (Sivertsen, Hassel, and Uhlig, unpublished), so the inhibitory concentrations in vitro are just within the range of relevant in vivo concentrations. In contrast, both mercaptopropionoic acid and mercaptobutyric acid show significant inhibition of GAD in vitro already at concentrations of 0.1–0.5 mM (Lamar 1970). Neither variation in glutamate concentrations nor of pyridoxal phosphate concentrations in the assay changed the strength of the inhibitory effect. This indicates that the inhibition of GAD was not competitive with regard to substrate or coenzyme in the enzymatic process. This is in contrast to the effect of mercaptopropionic acid, which is clearly competitive with respect to glutamate (Lamar 1970). Our testing of other substituted imidazoles showed that they all had an inhibitory effect on GAD activity in the same range, with 2MeI giving the strongest inhibition of GAD. This is in contrast to experiments in vivo, where 1MeI and 2MeI show much weaker tremorogenic and convulsive activity than 4MeI (Nishie et al. 1969). Finally, lethal poisoning of mice with 4MeI did not change the cerebral levels of GABA, in strong contrast to the effect seen with mercaptopropionic acid (Karlsson et al. 1974). Taken together, these results do not confirm inhibition of cerebral GABA synthesis as a decisive mechanism in 4MeI toxicity.

In the GABA receptor binding experiments, 4MeI was found to inhibit specific binding of [^3^H]GABA to cerebral GABA receptors, but the inhibition was statistically significant only in concentrations at 5 mM or above, and 50 mM was needed to inhibit the GABA receptor binding with 76%. As mentioned, these concentrations are higher than the whole brain concentrations of 4MeI after lethal poisoning. Therefore, also the inhibition of GABA receptor binding seems too weak to be a main mechanism in 4MeI CNS toxicity.

In conclusion, the results of this study do in our opinion indicate that neither 4MeI inhibition of GABA synthesis nor interference with specific GABA receptor binding in mouse brain provide a satisfactory explanation of the convulsive effect of 4MeI in mice.

As mentioned in the introduction, Nishie et al. (1969) found that the convulsive effect of 4MeI could be counteracted by chlordiazepoxide and by sodium phenobarbital. Although benzodiazepines and barbiturates are known to act via the GABA_A_ receptor, both groups have a clinically broad anticonvulsive effect, counteracting seizures induced by different agents and pathological mechanisms (McNamara 2006). The possibility that the convulsive effect of 4MeI is related to interference with cerebral GABA activity cannot yet be finally excluded, as some GABA receptor inhibitors like picrotoxin bind to other receptor sites than GABA itself (Olsen and Leeb-Lundberg 1981). Still, our results do indicate that the effect of 4MeI on other receptor systems in the mammalian brain should be investigated.
